# Live Birth from Slow-Frozen Rabbit Oocytes after *In Vivo* Fertilisation

**DOI:** 10.1371/journal.pone.0083399

**Published:** 2013-12-17

**Authors:** Estrella Jiménez-Trigos, José S. Vicente, Francisco Marco-Jiménez

**Affiliations:** Institute of Science and Animal Technology, Laboratorio de Biotecnología de la Reproducción, Universitat Politècnica de València, Valencia, Spain; Institute of Zoology, Chinese Academy of Sciences, China

## Abstract

*In vivo* fertilisation techniques such as intraoviductal oocyte transfer have been considered as alternatives to bypass the inadequacy of conventional *in vitro* fertilisation in rabbit. There is only one study in the literature, published in 1989, that reports live offspring from cryopreserved rabbit oocytes. The aim of the present study was to establish the *in vivo* fertilisation procedure to generate live offspring with frozen oocytes. First, the effect of two recipient models (i) ovariectomised or (ii) oviduct ligated immediately after transfer on the ability of fresh oocytes to fertilise were compared. Second, generation of live offspring from slow-frozen oocytes was carried out using the ligated oviduct recipient model. Throughout the experiment, recipients were artificially inseminated 9 hours prior to oocyte transfer. In the first experiment, two days after unilateral transfer of fresh oocytes, oviducts and uterine horns were flushed to assess embryo recovery rates. The embryo recovery rates were low compared to control in both ovariectomised and ligated oviduct groups. However, ligated oviduct recipient showed significantly (*P*<0.05) higher embryo recovery rates compared to ovariectomised and control-transferred. In the second experiment, using bilateral oviduct ligation model, all females that received slow-frozen oocytes became pregnant and delivered a total of 4 live young naturally. Thus, *in vivo* fertilisation is an effective technique to generate live offspring using slow-frozen oocytes in rabbits.

## Introduction

Storage below the critical temperature of −130°C allows the preservation of cells and tissues after a long storage in liquid nitrogen (LN2) without any decrease in viability. [1], [2]. There are currently two methods for gamete and embryo cryopreservation: slow-freezing and vitrification. The first to be developed was slow-freezing [3]. In this technique, germplasm is gradually exposed to low concentrations of cryoprotectants, in combination with very slow cooling rates, which leads to crystallization of extracellular water, resulting in an osmotic gradient that draws water from the intracellular compartment till intracellular vitrification occurs [4].

Since Whittingham [3] successfully froze mouse embryos, cryopreservation methodology have progressed to increase the number of lines, breeds and species that can be cryostored to preserve animal breeding and laboratory products (transgenics, clones) against loss caused by disease or hazards or improve the reproductive rate. For example, the rabbit breeding industry is increasingly using selected lines; the generation and characterisation of these lines require great effort and they must be kept in stock even if not needed for commercial use [5]. From a genetic standpoint, the cryopreservation of inbred strains is useful to establish control populations to study the genetic drift and gain when selection programmes are applied [5], [6], [7]. Although several breakthroughs have been made in oocyte cryopreservation since 1971, live offspring have only been obtained in a few species, such as mouse [8], human [9], rabbit [10], cattle [11], rat [12], horse [13] and cat [14]. Moreover, procedures developed for one species are difficult to adapt to another [15]–[19]. Specifically, few works have been carried out in rabbit [10], [20]–[28] and to the best of our knowledge, only Al-Hasani et al. [10] obtained live offspring from cryopreserved rabbit oocytes.

Rabbit has been used as an animal model organism to study mammalian reproduction for over a century [29], [30]. To this end, different technologies for *in vitro* development of rabbit embryos have been assayed, such as *in vitro* fertilisation (IVF) [31], [32], intracytoplasmic sperm injection (ICSI) [23], [33]–[36], and parthenogenetic activation [24], [26], [37]–[39]. Although it seems possible, IVF has not been successful in rabbit and a repeatable IVF technique has not yet been developed, possibly due to the lack of an efficient *in vitro* capacitation system for rabbit spermatozoa linked to the poor permeability of sperm plasma membrane [40]. Similarly, ICSI has been widely used in rabbit to study oocyte fertilisation and embryo development [33], [36]. However, this technique is difficult to perform because rabbit oocytes have rough, dark granules in the plasma and easily lyse and die after the ICSI process [23]. The success rate of rabbit ICSI is less than 4% [34], [35]. Some studies have employed parthenogenetic activation technique as an alternative tool to evaluate oocyte competence and *in vitro* development in rabbit [24], [26]. However, one substantial limitation to this technique is that it cannot obtain offspring. Thus, *in vivo* techniques, such as intraoviductal oocyte transfer and intrafollicular oocyte transfer, have been considered as alternatives to bypass the inadequacy of conventional *in vitro* fertilisation techniques [41], [42].

The aim of the present study was to develop a reliable and reproducible technique to generate live rabbit offspring with frozen oocytes.

## Materials and Methods

All chemicals and reagents were purchased from the Sigma-Aldrich Corporation (St. Louis, MO, USA) unless otherwise stated. The study was approved by the ethical committee of the Universidad Politécnica de Valencia. All animals were handled according to the principles of animal care published by Spanish Royal Decree 53/2013 (BOE, 2013; BOE  =  Official Spanish State Gazette).

### Animals

We used New Zealand White females (5 months old) for the collection of metaphase II (MII) oocytes and New Zealand White males (8 months old) for artificial insemination (AI). All the animals used as donors and recipients in this study belonged to a selected line based on New Zealand White rabbits selected since 1980 by a family index for litter size at weaning. The animals used came from the experimental farm of the Universidad Politécnica de Valencia. The rabbits were housed in conventional housing (with light alternating cycle of 16 light hours and eight dark hours, under controlled environmental conditions: average daily minimum and maximum temperature of 17.5 and 25.5°C, respectively), using individual cages (700×500×320 mm) with free access to a commercial diet and filtered water.

### Animal models: unilateral ovariectomised and unilateral oviduct ligation

Unilateral ovariectomy technique: Females had surgery before puberty (at 16 weeks of age). Animals were sedated by intramuscular injection of 16 mg xylazine (Rompun, Bayer AG, Leverkusen, Germany). As surgical preparation for laparotomy, anaesthesia was performed by intravenous injection of 16–20 mg ketamine hydrochloride (Imalgene®, Merial, S.A., Lyon, France) into the marginal ear vein. During laparoscopy, 12 mg of morphine hydrochloride (Morfina®, B.Braun, Barcelona, Spain) was administered intramuscularly. After surgery, does were treated with antibiotics (200,000 IU procaine penicillin and 250 mg streptomycin, Duphapen® Strep, Pfizer, S.L.) and buprenorphine hydrochloride (0.08 mg every 12 hours for 3 days, Buprex®, Esteve, Barcelona, Spain). Ovariectomy was performed gripping the left ovary with haemostatic tongs; blood vessels were ligated avoiding the oviduct and the ovary was removed. Abdominal wall and skin were closed using absorbable suture material (Monosyn®, B. Braun, Spain).

**Figure 1:**
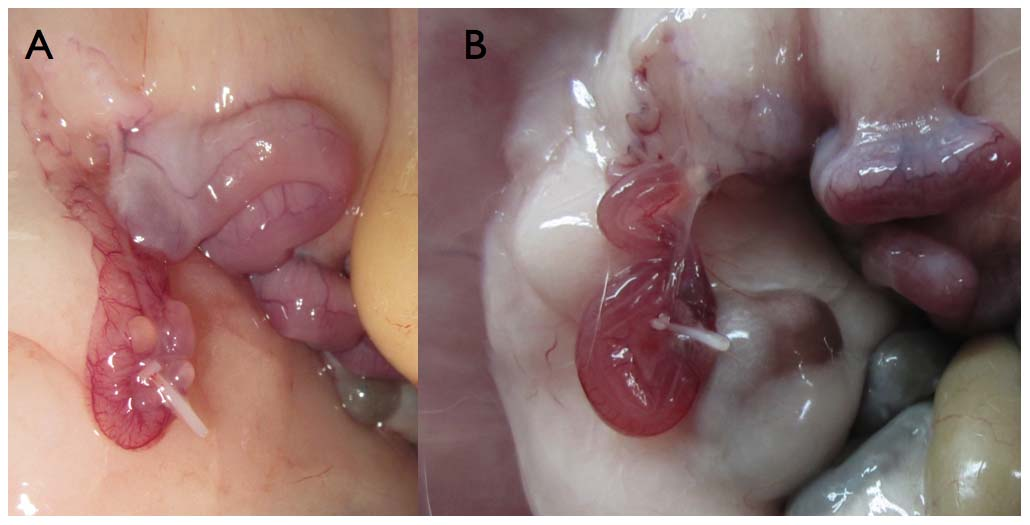
Representative oviduct ligation with a non-absorbable polymer locking clip (Hemo-o-lock® Ligation System) after transfer (48 h post ovulation induction) (A). Detail of intrauterine fluid retention after transfer (48 h post ovulation induction) (B).

Ligated oviduct assisted by laparoscopy: The equipment used was a Hopkins® Laparoscope, which is a 0°-mm straight-viewing laparoscope, 30-cm in length, with a 5-mm working channel (Karl Storz Endoscopia Ibérica S.A. Madrid). Recipients were anaesthetised as previously. The abdominal region was shaved, and the animals were then placed on an operating table in a vertical position (head down at 45-degree angle). This vertical positioning ensures that the stomach and intestines are cranially located so that the Fallopian tubes form a downwardly pointing loop between the ovaries and uterus. The endoscope trocar and traumatic forceps were inserted into the abdominal cavity. When the trocar was removed, the abdomen was insufflated with CO_2_ and the endoscope was then inserted. Oviduct was closed with a non-absorbable polymer locking clip (Hem-o-lok® Ligation System), applied by laparoscopy using a 5 mm automatic endoscopic locking clip applier (Reflex® Clip Applier, Conmed® Corporation, Utica, USA). Hem-o-lok product line is a ligation system that allows suture of 2 mm to 16 mm of vessel and/or tissue bundle. The clip was placed at the ampulla to prevent entry of the recipient's own oocytes (Figure 1A).

### Oocyte recovery

Donor females were induced to ovulate by intramuscular dose of 1 µg of Buserelin Acetate (Suprefact, Hoechst Marion Roussel, S.A., Madrid, Spain) [43]. Oocytes were collected from the oviducts 14–15 h after ovulation induction by flushing each oviduct with Dulbecco's phosphate-buffered saline without calcium chloride (DPBS) and supplemented with 0.1% of bovine serum albumin (BSA). Cumulus cells were removed and oocytes were incubated for 15 min at room temperature with 0.1% hyaluronidase.

### Intraoviductal oocyte transfer

Recipient females were inseminated 9 h prior to transfer with 0.5 mL of fresh heterospermic pool semen at a rate of 40×10^6^ spermatozoa/mL in Tris-citric-glucose extender [44]. The semen collection method was carried out using an artificial vagina, as described by Vicente et al. [43]. Motility was examined at room temperature under a microscope with phase-contrast optics at 40× magnitude. Only those ejaculates with >70% motile sperm (minimum requirements commonly used in artificial insemination) were pooled [45]. Immediately after insemination, ovulation was induced by an intramuscular injection of 1 µg Buserelin Acetate.

A detailed description of the technique used in rabbit was published previously [46]. Laparoscopic oviductal transfer was performed as previously described, but using only the endoscope trocar. For oocyte transfer, oocytes were aspirated in an epidural catheter (Vygon corporate, Paterna, Valencia), introduced into the inguinal region with an epidural needle and then inserted in the oviduct through the infundibulum. Transfers were always done unilaterally into the left oviduct, while the right oviduct was used as control. Prior to transfer, it was confirmed that ovulation had not yet taken place. In the ligation group, just after transfer the oviduct was ligated. Finally, the peritoneal air was removed from the abdominal cavity, the incision was sprayed with a Dermafill plastic dressing (Nobecutan, Laboratorios Inibsa, S.A. Barcelona) and antibiotic was intramuscularly administered (200,000 IU procaine penicillin and 250 mg streptomycin, Duphapen® Strep, Pfizer, S.L.).

### Slow-freezing protocol

The slow-freezing procedure was adapted from previously described methods [22]. Briefly, oocytes were incubated for 15 min at room temperature in a solution containing 1.5 M 1,2-propanediol (PROH) in DPBS and 20% FBS. Oocytes were then placed for 10 min in the freezing solution composed of 1.5 M PROH and 0.2 M sucrose in DPBS and 20% FBS and mounted between two air bubbles in 0.25-ml sterile French mini straws (IMV Technologies. L'Aigle, France) sealed by a sterile plug. The straws were then placed in a programmable freezer (Cryologic, CL-8800) for the freezing process. Temperature was lowered from 20°C to −7°C at a rate of 2°C/min. Manual seeding was performed at −7°C. Temperature was then lowered to −30°C at a rate of 0.3°C/min. Finally, straws were directly plunged into LN2 and stored for later use.

For thawing, the straws were taken out of the LN2 into ambient temperature for 10–15 s and plunged into a 20°C water bath. Oocytes were transferred stepwise into decreasing sucrose solutions (0.5, 0.3 and 0.1 M sucrose in TCM199 with 20% FBS) for 5 min before being equilibrated for 10 min in TCM199 containing 20% (v/v) FBS. Then, the oocytes were incubated for 2 h in medium TCM199 containing 20% (v/v) Foetal Bovine Serum (FBS) at 38.5°C and 5% CO2 in humidified atmosphere.

## Experiment Design

### Experiment 1. I*n vivo* fertilisation of fresh oocytes

**Figure 2:**
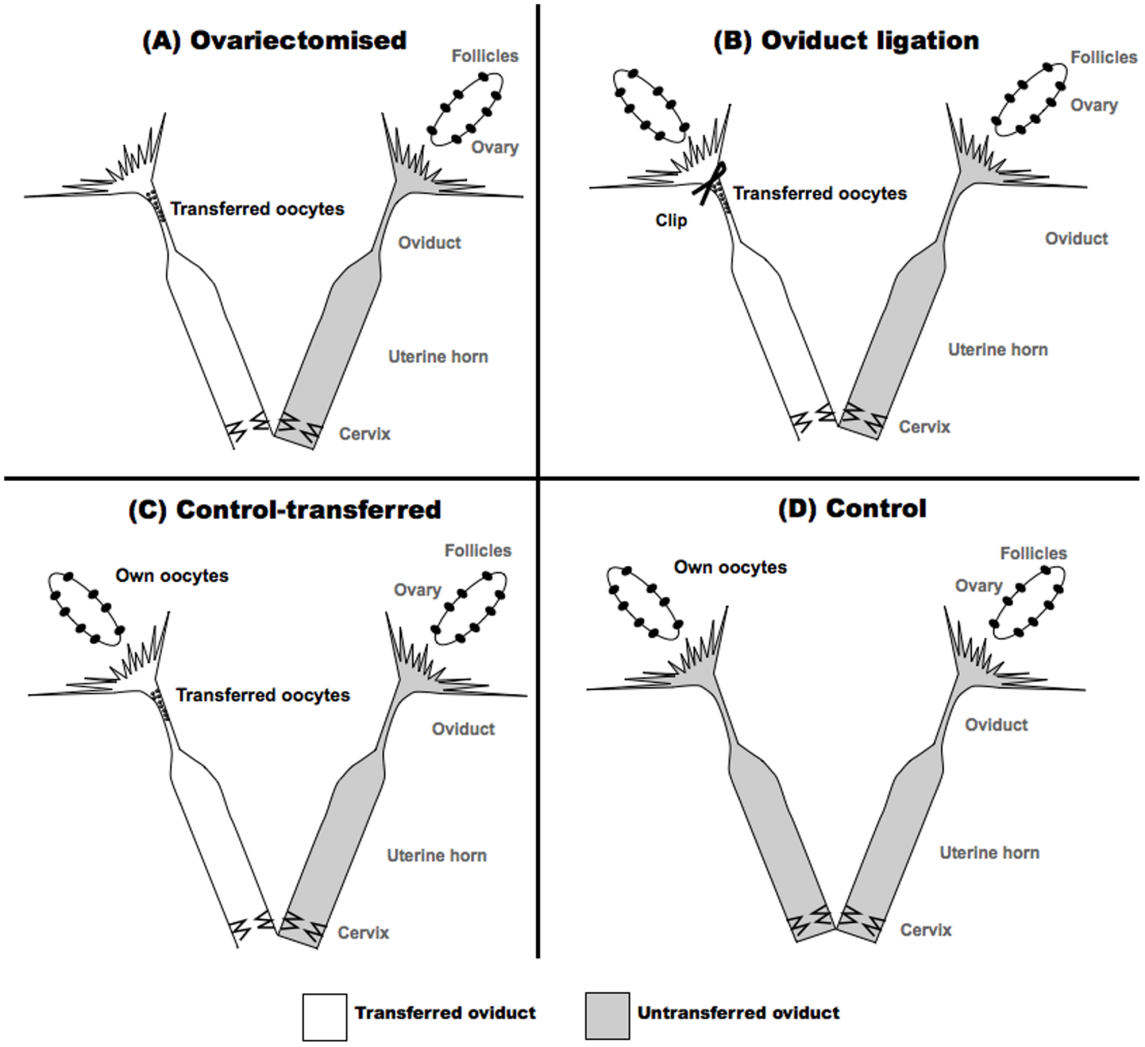
Experimental design of *in vivo* fertilisation in rabbit after intraoviductal transfer of oocytes into (A) unilateral ovariectomised, (B) unilateral oviduct ligated (oviduct was immediately ligated after oocytes transfer), (C) control-transferred and (D) control (no oocytes transferred) females. All transfers were always done unilaterally (left oviduct). Between 10 and 20 oocytes were transferred per oviduct, except to control females.

Females were divided into 4 groups: unilateral ovariectomy (n = 4), unilateral ligated oviduct (n = 8), control-transferred (n = 6) and control (n = 6) (Figure 2). The number of transferred oocytes per female varied from 10 to 20 (normal proportion of oocytes obtained after a superovulation treatment [47]), depending on the number of oocytes available in each session (5 sessions were performed). Two days after insemination, oviducts and uterine horns were removed and each was flushed separately with 5 ml of DPBS containing 0.1% of BSA to assess the embryo and oocyte recovery rates.

To discard any sperm effect, a sample of pooled embryos and oocytes from all transfer groups and sessions were fixed in DPBS containing 4% (w/v) buffered neutral paraformaldehyde solution for 2 h at room temperature. Then, embryos and oocytes were placed into 500 µl drops of DPBS containing Hoechst 33342 (*2′-(4-Ethoxyphenyl)-5-(4-methyl-1-piperazinyl)-2,5′-bi-1H-benzimidazol trihydrochloride*; 6 µM) and incubated for 15 min at room temperature in darkness. Embryos and oocytes were then washed twice, mounted, and the spermatozoa binding to the zona pellucida counted under a microscope equipped with ultraviolet illumination (excitation at 330–380 nm, emission at 460 nm).

### Experiment 2. Generation of live offspring from slow-frozen oocytes

To generate live offspring, after 9 hours of insemination a total of 121 slow frozen and thawed oocytes classified as normal (homogeneous cytoplasm, no vacuoles or granulations and an intact zona pellucid) and 38 fresh oocytes were transferred into both oviducts by laparoscopy to 6 recipient does (15 to 30 oocytes per recipient) and later oviducts were closed with a non-absorbable polymer locking clip (assessed in the results of experiment 1). Survival rates of slow-frozen oocytes were assessed by laparoscopy in the recipient does on the basis of implantation rate (number of implanted embryos at day 14 from total oocytes transferred) and birth rate (kits born/total oocytes transferred). To prove the sterility of oviduct ligated recipients, females were inseminated at day 21 postpartum and evaluated the implantation rate fourteen days later.

### Statistical analysis

To compare recovery rates and embryo recovery rates according to the intraoviductal transfer model (unilateral ovariectomised, unilateral oviduct ligation, control-transferred and control, experiment 1) as a fixed factor and the type of oocytes (fresh and slow-frozen, experiment 2) on implantation and offspring at birth as a fixed factor, a generalised linear model was used. The error was designated as having a binomial distribution using the probit link function. Binomial data for hatching or hatched blastocyst rate were assigned a value of one if positive development had been achieved or a zero if it had not. *P*<0.05 was considered significant. Data are shown as means ± standard error of means (S.E.M.). All analyses were performed with SPSS 16.0 software package (SPSS Inc., Chicago, Illinois, USA, 2002).

## Results

A total of 9 females were ovariectomised, but at time of transfer the epidural needle could only be inserted into the oviduct through the infundibulum of 4 females. Although 12 females underwent oviduct ligation, only 8 rabbits were included in the experiment, because after euthanasia 4 females presented tubal fluid accumulation and were rejected (Figure 1B). Only the intact females were considered in the following analysis (4 and 8 females for ovariectomised and oviduct ligation groups, respectively).

**Figure 3:**
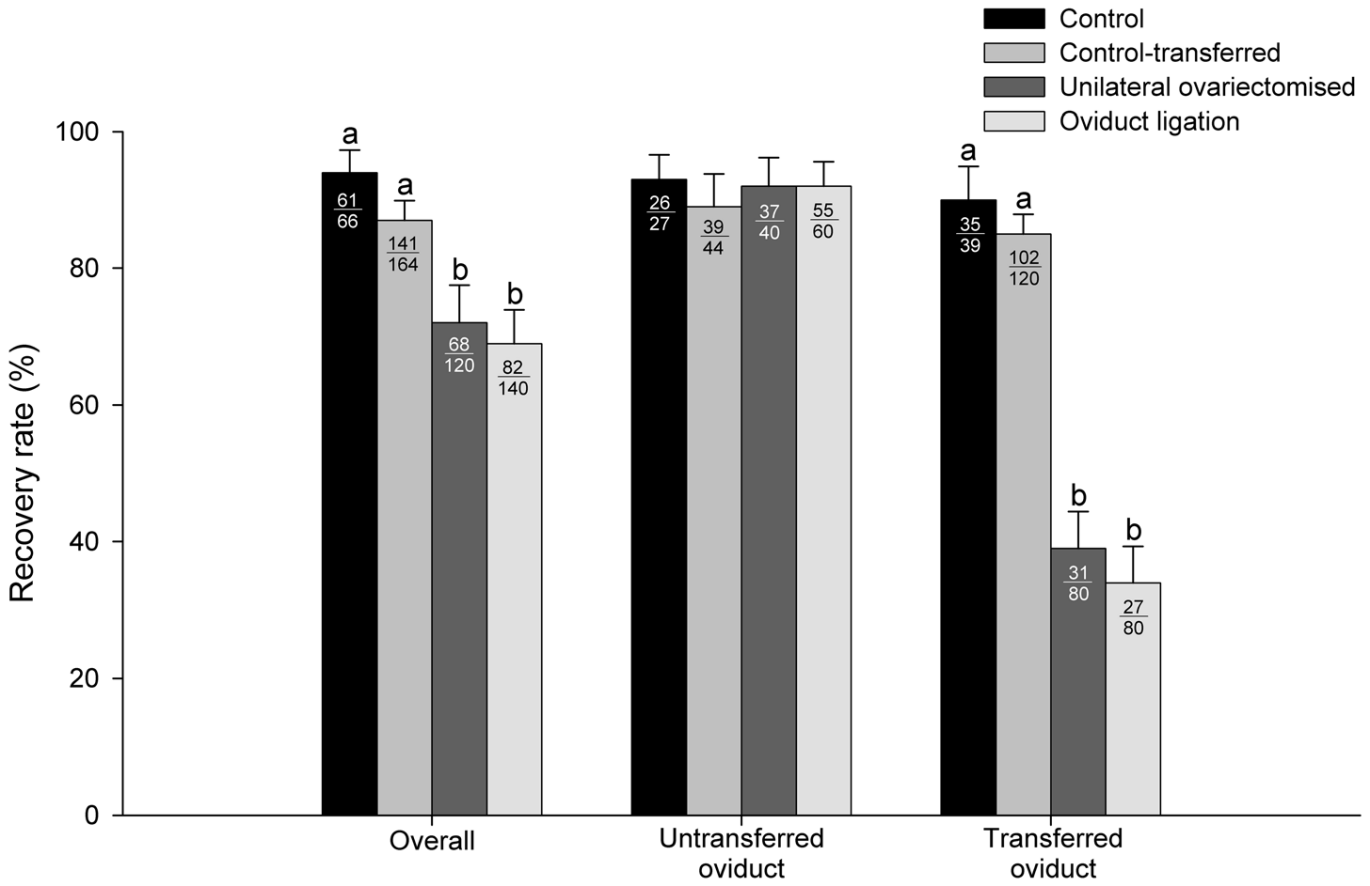
Overall recovery rate of *in vivo* fertilisation in rabbit after unilateral intraoviductal transfer of oocytes into ovariectomised, oviduct ligated, control-transferred (recovery rates calculated in excess to the number of ovulations) and control (no oocytes transferred) females. The numbers inside the bars indicate the number of oocytes and embryos recovered/total. Bars with different superscripts denote statistically significant differences between groups (P<0.05). Data shown are representative of five independent sessions.

**Figure 4:**
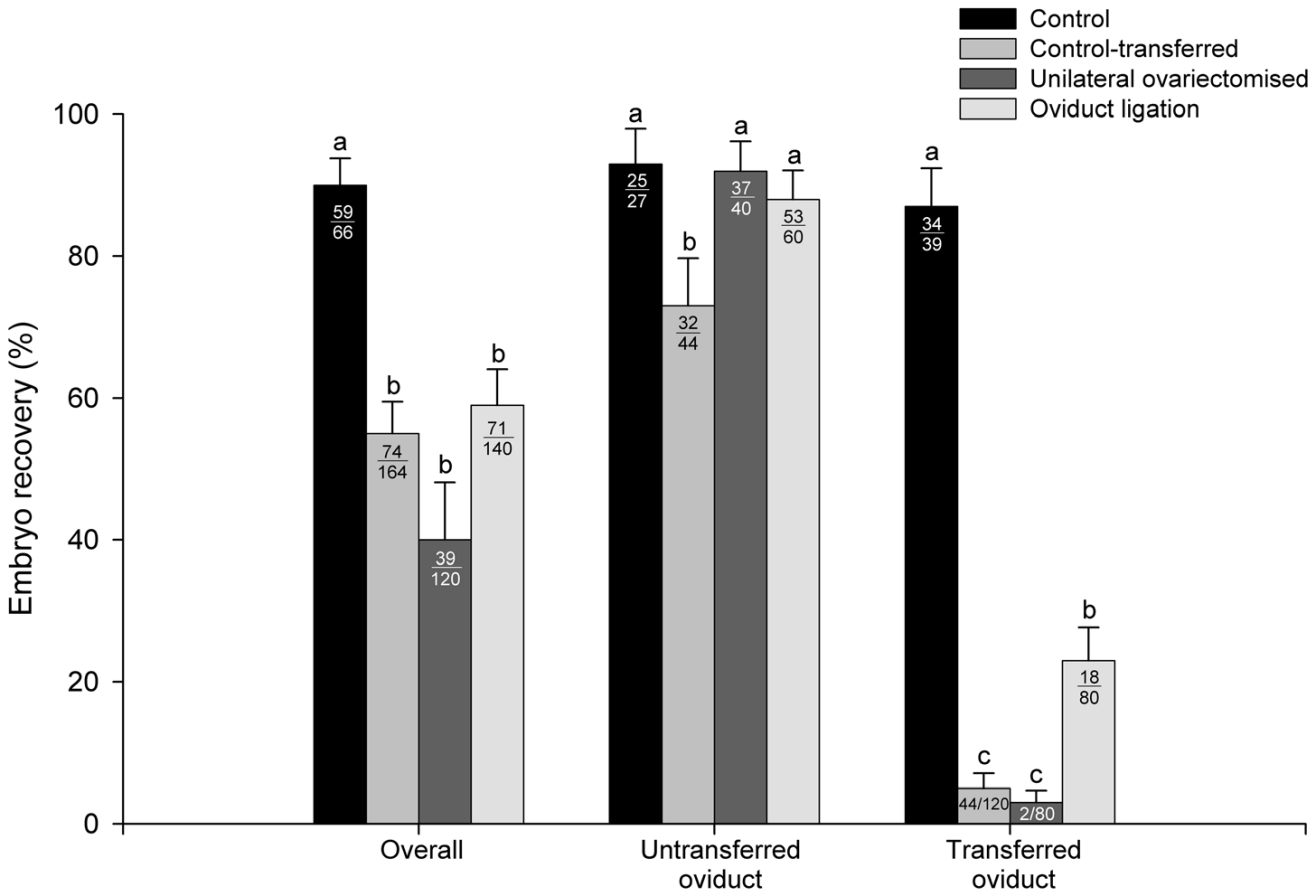
Embryo recovery rate of *in vivo* fertilisation in rabbit after unilateral intraoviductal transfer of oocytes into ovariectomised, oviduct ligated, control-transferred (recovery rates calculated in excess to the number of ovulations) and control (no oocytes transferred) females. The numbers inside the bars indicate the number of embryos recovered/total. Bars with different superscripts denote statistically significant differences between groups (P<0.05). Data shown are representative of five independent sessions.

All recovered embryos and oocytes were from oviducts and none were found in uterine horns. Overall recovery rates in ovariectomised and ligated females were significantly lower than in control groups (69.0±4.90% and 72.0±5.50% *vs* 87.0±2.90% and 94.0±3.30%, for ligated and ovariectomised *vs* control-transferred and control, respectively, Figure 3). Likewise, an overall reduction in embryo recovery rate was observed in all transferred groups (40.0±8.10%, 55.0±4.50%, 59.0±5.10% and 90.0±3.80%, for unilateral ovariectomy, control-transferred, ligated and control, respectively, Figure 4). When oviducts were analysed separately, in untransferred oviduct similar recovery rates were observed in all of the experimental groups (Figure 3). However, in transferred oviduct, the recovery rate in ovariectomised and ligated females was significantly lower than in both control groups (34.0±5.30%, 39.0±5.40%, 85.0±2.90% and 90.0±4.90%, for ligated, ovariectomised, transferred-control and control, respectively, Figure 3). Likewise, embryo recovery rates in untransferred oviduct were similar for all experimental groups, except for the control-transferred group (93.0±5.00%, 92.0±4.20%, 88.0±4.10% and 73.0±6.70%, for control, ovariectomised, ligated, and transferred-control, respectively, Figure 3). However, in transferred oviduct, embryo recovery rates in ovariectomised, control-transferred and ligated were significantly lower than in control (3.0±1.70%, 5.0±2.10%, 23.0±4.70% and 87.0±5.4%, respectively, Figure 4), despite the fact that embryos produced after intraoviductal transfer and the control embryos presented similar numbers of spermatozoa binding to the zona pellucida per embryo (13.7±1.71 and 17.7±1.58, control-transferred and control, respectively). In line with this result, oocytes that failed in fertilisation showed similar numbers of spermatozoa binding to the zona pellucida per oocyte, independently of the experimental group (4.2±2.99 and 3.0±8.20, for control-transferred and control, respectively).

**Figure 5:**
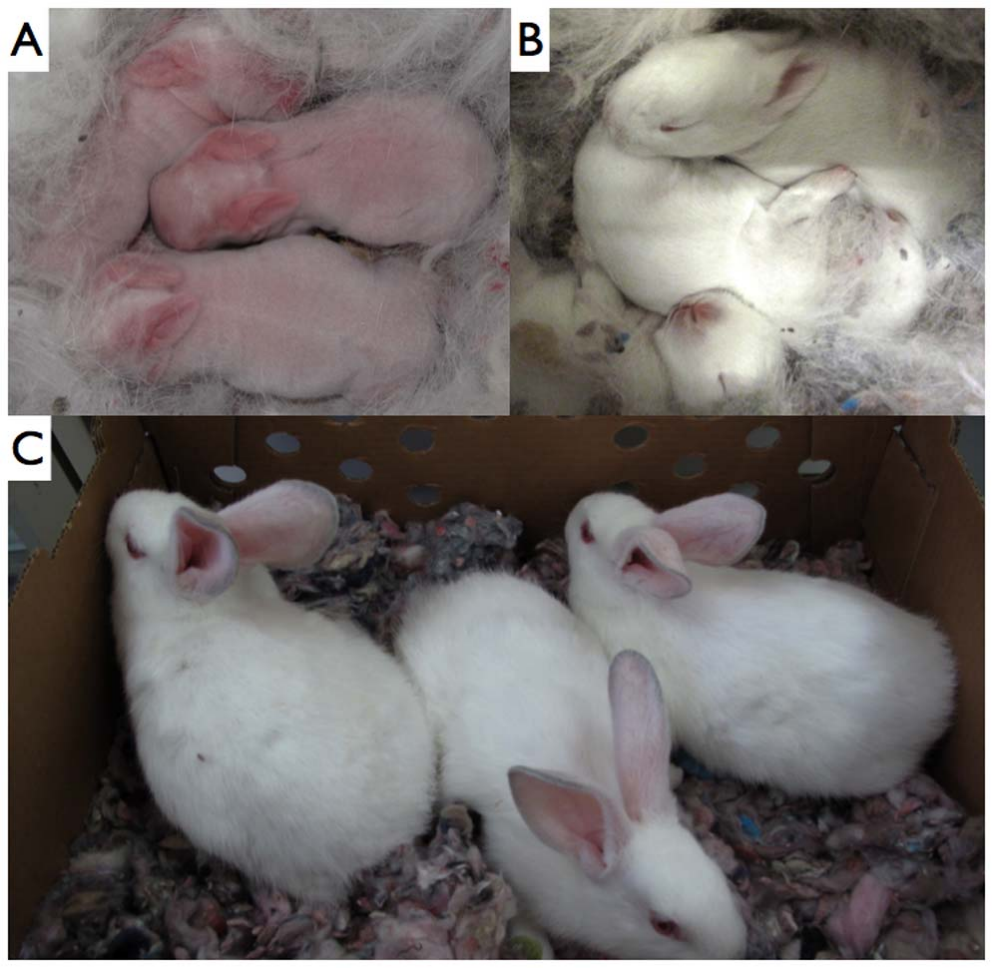
Rabbit derived from oocytes cryopreserved with slow-freezing protocol. (A) After birth (B), at 21 days old and (C) at 71 days old.

**Figure 6:**
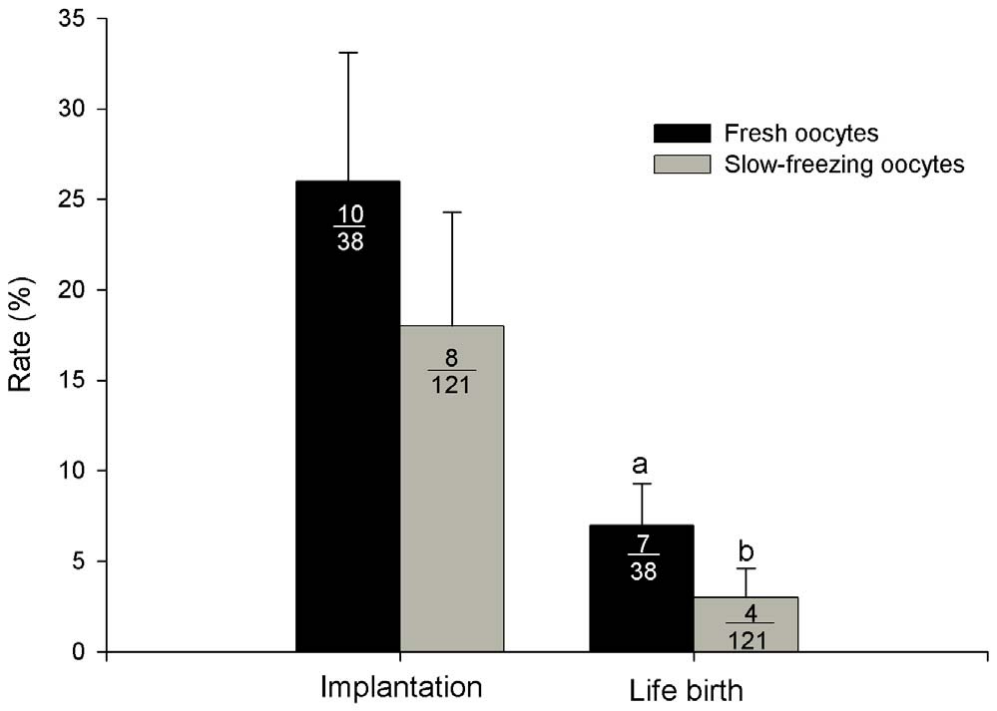
*In vivo* development of slow-frozen oocytes from rabbit. The numbers inside the bars indicate the number of oocytes transferred/total for implantation and life birth rates. Bars with different superscripts denote statistically significant differences between groups (P<0.05).

All transferred females that received cryopreserved oocytes became pregnant, and delivered a total of 4 live young naturally; 3 of these kits presented survival and growth until weaning at approximately 70 d of age (Figure 5). The offspring were visually normal. In the fresh transferred oocytes group, the implantation rate was 26.0±7.1%, while in the slow-frozen oocytes group, the implantation rate was 7.0±2.3% (Figure 6). The overall rate of offspring obtained using slow-frozen oocytes was significantly lower (3.0±1.6% vs. 18.0±6.3% for slow-frozen vs. fresh oocytes, respectively, Figure 6). None of the oviduct ligated recipients inseminated at day 21 postpartum had implanted embryos.

## Discussion

Historically, the rabbit was a ‘classic’ species in the early decades of embryology and reproductive biology, starting from the late 19th century [48]. However, although it seems possible, the use of assisted reproductive technology has not been successful in rabbit and a repeatable IVF or ICSI techniques has not yet been developed [33, 36 40]. We performed *in vivo* experiments in the rabbit for two reasons: its reproductive features allowed us to manage oocyte transfer according to the timing of gamete biological events and to mimic the uterine environment to improve fertilisation and the embryo culture systems. To date, *in vitro* conditions have been unable to mimic the dynamic changes of oviduct and uterus secretion that respond to the varying metabolism of a developing embryo [49]–[53]. However, performing both animal models affected the reproductive tract functionality of half of the females. Thus, the entrance to the infundibulum was not accessible via laparoscopy as a consequence of the surgery in ovariectomised females, whereby oviduct ligation induced an accumulation of tubal fluid. This accumulation of tubal fluid after oviduct ligation has been described previously [54].

The oviduct plays a major part in different reproductive processes, providing the microenvironment for numerous steps in early embryogenesis [55].The overall recovery rate was similar between control-transferred and control group, in line with those reported in the literature [47]. Nevertheless, a low recovery rate was observed after transfer into unilateral ovariectomy and ligated group. This would indicate a tubal disorder in these models. Although this hypothesis is difficult to verify, it is based on the differences observed between control-transferred group and ovariectomy and ligated females. The low recovery rate during the first day after transfer has been observed previously [56], [57]. However, as high recovery rates were obtained in our control-transferred and control groups, we ruled out a negative impact of technology transfer. Physiological properties of the oviduct involve a complex interaction of gamete transport and muscular, ciliar, secretory and adhesive functions [58]. Nevertheless, we ruled out an altered tubal migration because all uterine horns were perfused separately and no oocytes or embryos were collected. It is known that oocyte transport occurs in the opposite direction to the oviductal fluid current, which flows towards the peritoneal cavity, coinciding with the maximum secretion of oviductal fluid during oestrogen dominance [59], [60]. However, the oviduct ligated group ensures that no loss of oocytes occurs in the peritoneal cavity. Thus, the recovery rate obtained in ovariectomised females (“open” system) was similar to that reported in ligated does. Assuming that once oocytes are transferred to the oviduct they do not become lost in the peritoneal cavity or in the uterus, the issue remains of how they are retained by the oviduct in both models. Both groups have in common the absence of follicular fluid as opposed to control-transferred females. At least a part of this fluid must be transported into the oviduct together with the cumulus-oocyte complex [61]. Thus, the absence of the ovulation product could induce the retention oocyte in the oviduct. However, this hypothesis needs to be tested.

Overall embryo recovery rates from the untransferred oviduct for the all groups were similar. Therefore, synchronisation between artificial insemination and oocyte transfer was efficient. This conclusion is reinforced by the fact that embryos and oocytes failing to fertilise, regardless of the experimental group, presented similar numbers of sperm binding to the zona pellucida. Thus, we also confirm that anaesthetics did not affect the sperm transport [62]. However, in all transferred groups, embryo recovery rates decreased significantly. This result suggests that intraoviductal oocyte transfer reduced the probability of fertilisation. Some studies suggest that removal of cumulus cells prior to IVF reduced the cleavage rate through loss of a factor secreted by these cells [63]. It has also been suggested that cumulus cells continuously secrete sperm attractants [54]. Thus, capacitated spermatozoa are guided to reach the oocyte surface, passing through the cumulus mass. Moreover, our results demonstrated that despite similar numbers of spermatozoa binding to the zona pellucida, successful fertilisation was not achieved. Nevertheless, this hypothesis needs to be tested.

While numerous reports of studies designed to investigate oocyte cryopreservation have been published in some species [64], few works have been performed in rabbit [10], [20]–[28] and only two recent works compared slow-freeze and vitrification methods [24], [26]. Moreover, live offspring were obtained only in one report, using the slow-freezing method [10]. Rabbit oocytes are not very sensitive to low temperatures but present particularly sensitivity to high levels of cryoprotectants, and this has been shown to have a dramatic effect on the meiotic spindle configuration [20], [21], [23], [24], [26], [27]. To date, there are no reports of offspring obtained from vitrified rabbit oocytes. Although several strategies have been developed to improve cryopreservation results [65], our data would clearly suggest that there is a developmental advantage for slow-frozen oocytes being transferred for in vivo fertilisation and in vivo embryo development. Following oocyte transfer, there were significant differences in births per oocyte cryopreserved between fresh and slow-frozen oocytes (18% v 3%). However, our offspring rates were similar to those reported for oocytes cryopreserved (In human, [66], bovine, [67]–[69] and mouse [70]–[72]). Specifically, in rabbits using a slow-freezing method has resulted in live offspring with a total of 0.8% [10]. Our model may therefore have an advantage, since the *in vivo* environment provides optimal conditions that an *in vitro* assay is unable to provide.

Our results indicated that oviduct manipulation to prevent the entrance of oocytes into the oviduct of the female recipient compromised the use of the reproductive tract in a high percentage of females. Taken together, our results demonstrate that we succeeded for the second time in the cryopreservation of rabbit oocytes. In conclusion, a combination of in vivo fertilisation and slow-frozen oocytes might be a useful approach to generate live offspring in rabbit. Nevertheless, further studies should be done to improve the recipient model.
